# Supplementary figures and images for: Influence of the Infill Orientation on the Properties of Zirconia Parts Produced by Fused Filament Fabrication
Source: Materials (Basel). 2020 Jul 15;13(14):3158. doi: 10.3390/ma13143158 (PMC7411807; doi:10.3390/ma13143158)

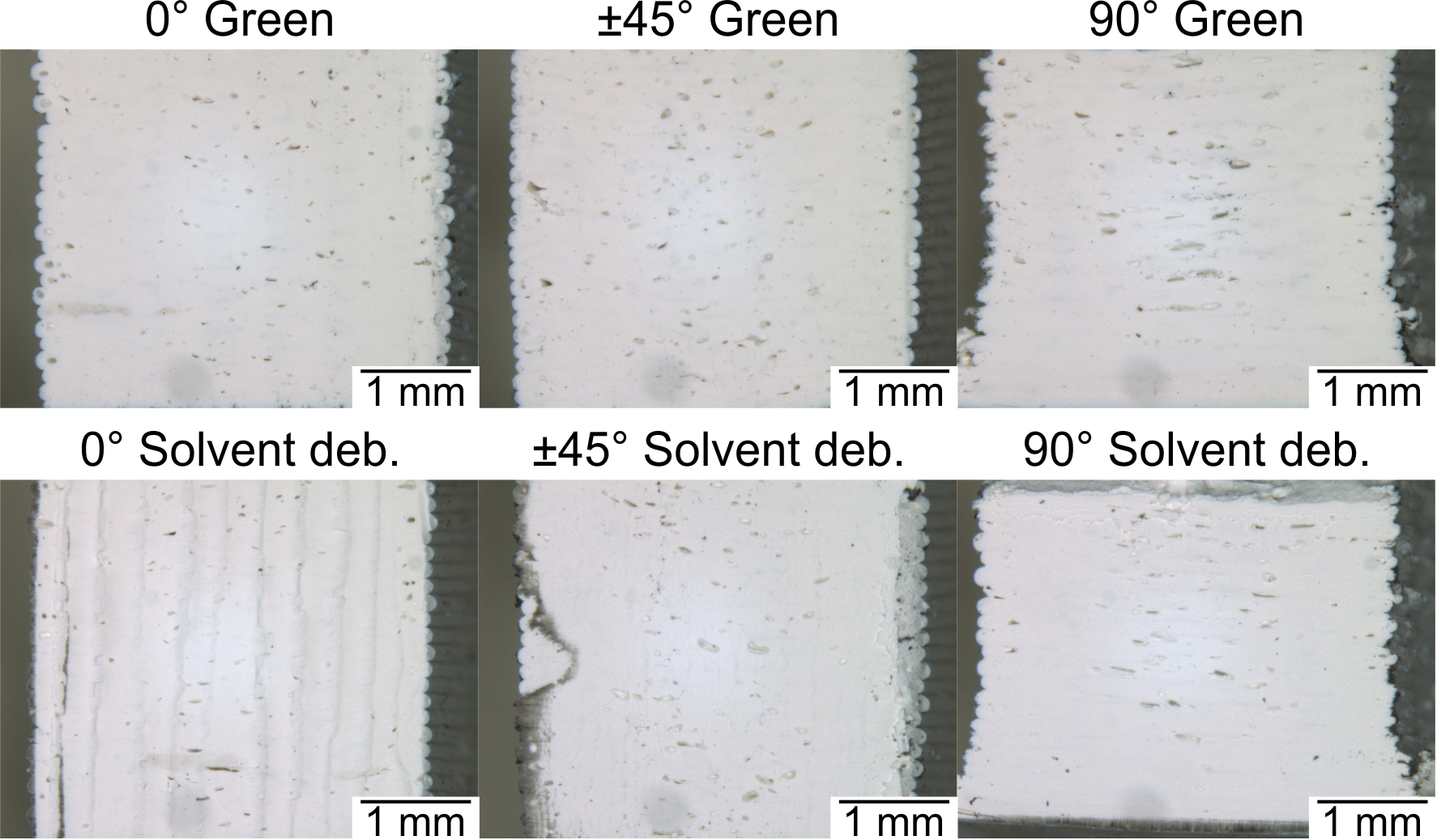

Supplement: Supplementary file 1 [file materials-13-03158-s001.zip › Supporting informaiton/Figure S1.jpg]

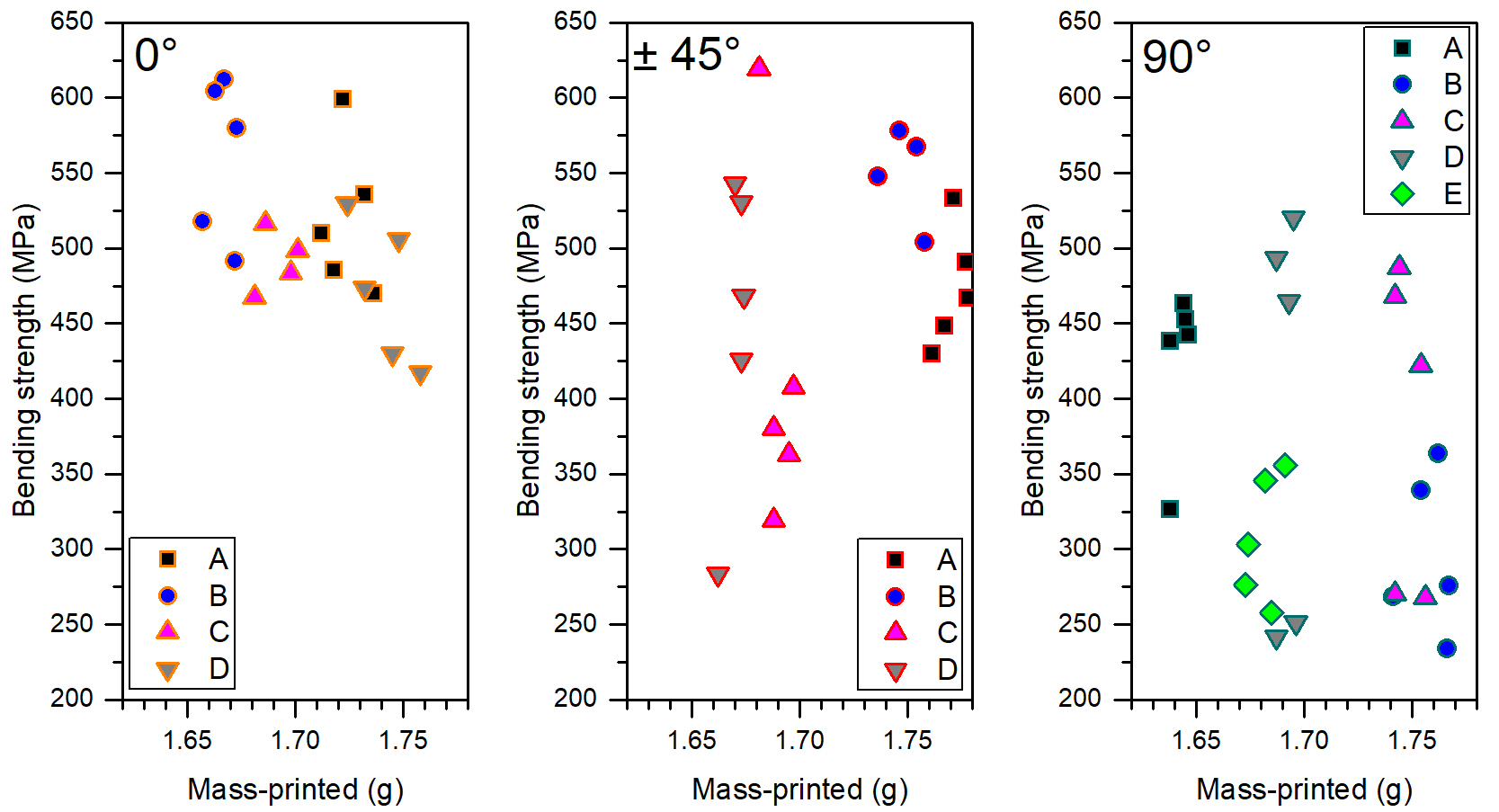

Supplement: Supplementary file 1 [file materials-13-03158-s001.zip › Supporting informaiton/Figure S2.jpg]
